# ApicoTFdb: the comprehensive web repository of apicomplexan transcription factors and transcription-associated co-factors

**DOI:** 10.1093/database/baz094

**Published:** 2019-09-16

**Authors:** Rahila Sardar, Abhinav Kaushik, Rajan Pandey, Asif Mohmmed, Shakir Ali, Dinesh Gupta

**Affiliations:** 1 Translational Bioinformatics Group, International Centre for Genetic Engineering and Biotechnology, Aruna Asaf Ali Marg, New Delhi 110067, India; 2 Parasite Cell Biology Group, International Centre for Genetic Engineering and Biotechnology, Aruna Asaf Ali Marg, New Delhi 110067, India; 3 Department of Biochemistry, Jamia Hamdard, Hamdard Nagar, New Delhi 110057, India

## Abstract

Despite significant progress in apicomplexan genome sequencing and genomics, the current list of experimentally validated transcription factors (TFs) in these genomes is incomplete and mainly consists of AP2 family of proteins, with only a limited number of non-AP2 family TFs and transcription-associated co-factors (TcoFs). We have performed a systematic bioinformatics-aided prediction of TFs and TcoFs in apicomplexan genomes and developed the ApicoTFdb database which consists of experimentally validated as well as computationally predicted TFs and TcoFs in 14 apicomplexan species. The predicted TFs are manually curated to complement the existing annotations. The current version of the database includes 1292 TFs which includes experimentally validated and computationally predicted TFs, representing 20 distinct families across 14 apicomplexan species. The predictions include TFs of TUB, NAC, BSD, HTH, Cupin/Jumonji, winged helix and FHA family proteins, not reported earlier as TFs in the genomes. Apart from TFs, ApicoTFdb also classifies TcoFs into three main subclasses: TRs, CRRs and RNARs, representing 2491 TcoFs in 14 apicomplexan species, are analyzed in this study. The database is designed to integrate different tools for comparative analysis. All entries in the database are dynamically linked with other databases, literature reference, protein–protein interactions, pathways and annotations associated with each protein. ApicoTFdb will be useful to the researchers interested in less-studied gene regulatory mechanisms mediating the complex life cycle of the apicomplexan parasites. The database will aid in the discovery of novel drug targets to much needed combat the growing drug resistance in the parasites.

## Introduction

Transcription regulation is a key process that facilitates the cellular responses to different environmental conditions. The underlying transcriptional machinery of regulation is more complex in eukaryotes as compared to that in prokaryotes due to involvement of a diverse set of transcriptional enzymes and proteins acting as regulators. These regulators consist of site-specific transcription factors (TFs) as well as general TFs (TBP, TFIIB, TFIIE and MBF) and specific RNA polymerases subunits (1). In general, eukaryotic genomes contain a large number of TFs, classified on the basis of more than 90 kinds of conserved DNA-binding domains (DBDs) (2). The TFs can bind to specific DNA sequences upstream of promoter regions, controlling the rate of transcription and thus transfer of genetic information (3, 4). Here, the sequence diversity among DBDs also ensures precise regulation of various cellular processes in response to external and internal perturbations (5). In fact, even in well-annotated organisms, numerous TFs have obscure DNA-binding sequences which can still direct complex transcription regulation (6). In addition, there are many proteins known as transcription-associated co-factors (TcoFs) which do not bind to DNA but interact with TFs for transcription regulations such as chromatin remodeling factor, also controlling the direction of gene regulation by assisting general TFs (7). Moreover, notwithstanding for the best-examined classes of DBDs, due to the diversity in protein as well as within the recognition sequences, the precise prediction of the regulators remains a challenging task (8).

This is especially important for recently sequenced genomes with several annotated proteins with unassigned functions, for example *Plasmodium* and other apicomplexans like *Eimeria*, *Theileria* and *Cryptosporidium* genomes. However, despite the need, the number of annotated TFs in these apicomplexan genomes is exceptionally limited as compared to model organisms like *Homo sapiens*, *Mus musculus* and *Arabidopsis thaliana* (9, 10). In general, identification of TFs is based either on the experimental findings, for instance ChIP-Seq and protein-binding microarrays (PBMs), or on computational analysis by exploiting traditional sequence similarity-based search, e.g. BLAST and HMMER (11). Herein, the computational methods compare the putative TF sequence with known DBDs as a reference for TF identification. However, several TFs share a low sequence similarity with known DBDs (11), making their identification and characterization a daunting task, using traditional methods alone.

Till date, a number of TF databases have been developed—AnimalTFDB for animals (12), PlantTFDB for plants (13), FlyTF for fruit flies (14) and TFCat (15) and TcoF-DB (16) for humans and mice—however, there is no report on any database dedicated to apicomplexan-specific TFs or their classified regulators. Using the *in silico* approach, Vaquero *et al.* identified 202 transcription-associated proteins in *Plasmodium falciparum* and classified them into general TFs, stage-specific TFs and chromatin-related proteins (17). However, only a limited number of TFs, mainly belonging to the AP2 family, are experimentally validated in the parasite (9). In virtue of their essential role in guiding the key cellular processes in the parasite’s life cycle involving multiple hosts, identification and characterization of novel TFs may provide deeper understanding of gene regulation in the parasite which may lead to identification of new drug targets. Therefore, in the present study, we performed an integrative *in silico* proteome analysis of 14 apicomplexan species in order to identify TFs and TcoFs, based on conserved DBD analysis, InterPro domain information (18) and gene ontology analysis (19). We are able to identify and report several new TFs and TcoFs belonging to diverse protein families among different apicomplexan species, including *P. falciparum*. Using this information, we have developed ApicoTFdb—a novel web-based repository for hosting the classified list of apicomplexan regulators and information related to their domain architecture, molecular function(s), biological pathway(s) and interologs dynamically linked to related literature. ApicoTFdb consists of 1292 TFs which includes experimentally validated and computationally predicted TFs from 20 distinct TF families and 2491 TcoFs for 14 apicomplexans. These apicomplexan parasites include parasite species which are causative agents of malaria, toxoplasmosis, babesiosis, cryptosporidiosis and poultry and cattle disease. We were also successfully able to provide functions to 322 proteins which were annotated as ‘hypothetical proteins’ or ‘proteins with unknown function’ in EuPathDB. With this database, we highlight several putative regulators that otherwise remain obscure with existing resources.

We believe that the presented database would be extremely useful for the scientific community interested in deducing the regulatory molecules and their mechanism that governs the complex life cycle of any of these 14 different apicomplexan parasites.

## Materials and methods

The protein sequences for TF identification across 14 apicomplexans species (see Table 1) were retrieved from PlasmoDB (version 38) (20), ToxoDB (version 38) (21), PiroplasmaDB (version 38) (22) and CryptoDB (version 38) (23). We also obtained the sequences representing different classes of DBDs from AnimalTFDB (version 2.0), DBD (Release 2.0) and footprintDB (as on 14 June 2018) (24). These TF sequences were used to create a reference database of TF-HMM profiles, scanned with the *hmm-search* program (HMMER version 3.1) (25). To determine the set of DBD-enriched protein sequences in the selected proteomes, we mapped each protein sequence to the reference HMM profiles (e-value < 0.001). Independently, we also searched conserved domains in each of the selected protein sequences using InterProScan 5 (version 5.31) (18). The results obtained from both of the independent methods were manually compared before assigning function to a given protein as putative TFs/TcoFs (details in next sections). The additional annotation for the assigned TFs/TcoFs, such as sequence length, gene and protein sequence, isoelectric point, molecular weight, previous IDs, and UniProt information were retrieved from EuPathDB. For each of the putative regulator, GO and biological pathway information was retrieved from AmiGO (26) and KEGG databases (27). The PPI information was retrieved from the STRING database (version 10.5) (28). Nuclear localization signals were predicted using NucPred (29), and CELLO2GO was used for subcellular localization prediction (30). PATS and the ApicoAP server were used to predict apicoplast-targeting sequences (31, 32).

**Table 1:** Comparison of ApicoTFdb transcription factor entries with those of existing databases, DBD and CisBP.

**Species**	**Genome size ( Mb)**	**Genome version** ^*^	**Total number of proteins**	**EuPathDB text search**	**Number of validated and predicted TFs in published databases**				**ApicoTFdb**			
						**DBD** ^**$**^		**CisBP** ^**#**^				
					**TF count**	**TF family**	**TF count**	**TF family**	**TF count**	**TF family**	**Experimentally verified**	**Unannotated proteins**
*P. falciparum*	23.3	2002-10-03	5635	548	18	7	44	10	96	16	27	7
*P. berghei*	18.5	2017-01-09	5089	418	19	12	46	11	92	18	13	9
*P. chabaudi*	18.8	2015-06-18	5282	372	19	7	46	11	95	18	13	12
*P. knowlesi*	24.3	2015-06-18	5261	386	14	8	46	10	79	16	12	5
*P. vivax*	28.8	2015-06-18	5390	372	15	7	49	8	93	17	12	30
*P. yoelii*	22.4	2005-09-01	7774	360	15	6	46	9	74	14	10	36
*T. gondii* ME49	64.5	2015-09-13	8920	443	N.A^+^	N.A^+^	81	14	146	17	1	12
*T. gondii p89*	64.1	2014-08-13	9874	486	N.A^+^	N.A^+^	N.A^+^	N.A^+^	145	16	0	4
*B. bovis*	8.1	2010-03-10	3721	126	N.A^+^	N.A^+^	N.A^+^	N.A^+^	33	6	0	11
*C. parvum*	9.1	2007-02-16	4020	248	30	11	35	8	73	14	1	4
*C. cayetanensis*	44	2016-09-22	7592	332	N.A^+^	N.A^+^	N.A^+^	N.A^+^	77	14	0	28
*N. caninum*	57.4	2015-02-21	7266	361	N.A^+^	N.A^+^	84	11	114	16	2	65
*E. maxima*	49.9	2013-11-05	6249	241	N.A^+^	N.A^+^	N.A^+^	N.A^+^	74	16	0	46
*E. tenella*	51.8	2013-11-05	8634	307	N.A^+^	N.A^+^	N.A^+^	N.A^+^	101	13	0	53
	Total no of TFs = 1292											

^*^These are the genome versions available on EuPathDB Release 38.

^$^DBD—transcription factor prediction database (Release 2.0).

^#^CisBP—Catalog of Inferred Sequence Binding Preferences (last updated: 5 April 2015, Database Build 1.02).

N.A.^+^—no information available.

### TF family assignment

**Figure 1:**
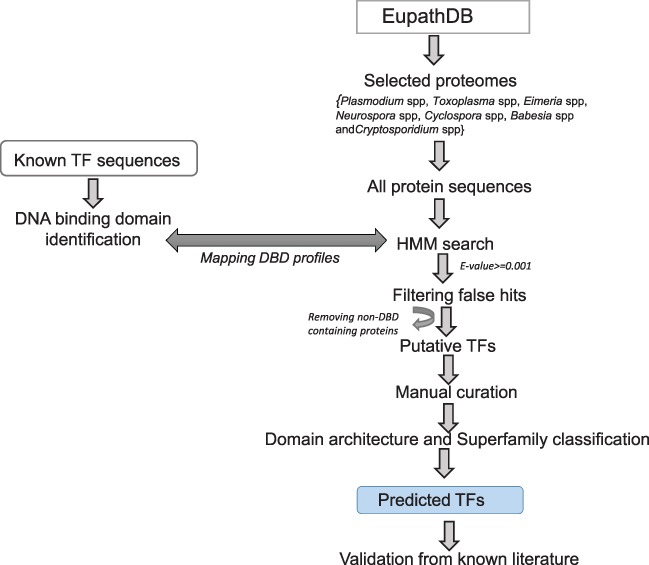
Methodology used for the prediction of TFs.

In order to classify a given protein sequence into a TF family, we exploited its DBD profiles using the methods mentioned before. For a given protein, we independently obtained its domain information predicted with InterProScan 5 and GO-based biological function, if available. We performed careful manual curation for each sequence by assigning a TF family to it on the basis of conserved DBD as shown in Figure 1 and integrating the abovementioned sources of information. Since a protein sequence may have more than one DBD, therefore, for proteins with more than one DBD, we assigned the TF family on the basis of the superfamily with the lowest e-value. In order to validate assignment rules and prediction results, we scanned the list of previously known TFs in our classified list of TFs. The known list of TFs was obtained by reviewing the recently published literature.

### Transcription-associated co-factor predictions

For TcoF predictions, we performed GO-based analysis of each protein sequence to search for different classes of regulators, i.e. transcriptional regulators (TRs), chromatin regulators (CRRs) and RNA regulators (RNARs), among apicomplexans. For TR classification, GO terms with ‘transcription coactivator activity’, ‘transcription corepressor activity’, ‘transcription co-factor activity’ and ‘regulation of transcription’ were used. For CRR predictions, the GO terms used were ‘chromatin remodeling’, ‘regulation of chromosome’, histone modification ‘histone *ylation’, ‘histone.*ylase activity’ and ‘histone *transferase activity’, as reported by Zhang *et al.* (12), whereas for RNA regulators we used the GO terms ‘RNA-binding’, ‘regulation of transcription by RNA-polymerase’, ‘transcription-RNA dependent’ and ‘transcription initiation from RNA-polymerase’. There were many proteins which were having annotations such as tRNA-associated proteins and ribosome assembly-associated proteins in their GO terms and product description which were removed manually.

### Website design and implementation

The ApicoTFdb web interface has been designed with XHTML, CSS and JavaScript languages. CSS and JavaScript were used for tables and other visualization. In-house PERL scripts perform the database search queries and data retrieval. ApicoTFdb is integrated with a number of additional utilities which facilitates querying the database in more than one way, for instance BLASTn and BLASTp for homology (33, 34). Additionally, each entry in ApicoTFdb is dynamically linked to PubMed and Google Scholar for the associated literature search.

## Results

Using the *in silico* approach, we predicted and classified the TFs and TcoFs for 14 apicomplexans species. ApicoTFdb thus provides a unique platform to analyze several new classes of TFs/TcoFs not reported earlier in the parasite genomes.

### TF prediction and classification

#### TF identification using HMM-based DBD identification

To predict and classify TFs into their respective superfamily, we identified the conserved DBDs across proteomes of all the target species. In order to achieve the set task, we retrieved all the known DBD profiles from repositories such as AnimalTFDB with 55, DBD database with 147 and footprintDB with 92 profiles, which were used as reference TF-DBD profiles for apicomplexan TF identification (Table S1). These shortlisted models were then used to scan the DBDs within each of the 14 apicomplexan proteomes.

Hence, the abovementioned TF prediction pipeline and manual curation resulted in the identification of 1292 putative TF proteins representing 20 TF families in the 14 apicomplexan species (Table S2). The results include 529 *Plasmodium* species TFs, 73 *Cryptosporidium* species TFs and overall 690 TFs from *Toxoplasma* species, *Eimeria* species, *Cyclospora* species, *Neurospora* species and *Babesia* species, as shown in Table 1.

#### Comparison with the experimentally verified TFs

All the predicted TFs were manually curated and analyzed for their biological functions. Among the predicted list of TFs, we observed a large number of hypothetical proteins and proteins with unknown functions. Table 1 summarizes the total number of hypothetical/unknown proteins classified as TF in each of the apicomplexan species. Within our predicted set of TFs, we observed a large number of validated TFs (Table 1). Since we are able to retain majority of known TFs, we extended our analysis to classify this list of TFs according to their respective domains.

#### Genome-wide analysis of transcription factors in apicomplexans

Using the TF prediction pipeline, we have successfully assigned functions to 322 proteins, previously annotated as hypothetical, uncharacterized, unspecified product and conserved proteins with unknown function under a putative TF class according to their DBDs (Table S3).

**Figure 2:**
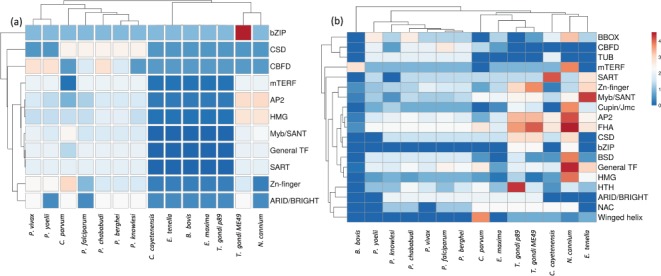
Heat map of TFs across the apicomplexans, analyzed in the study. Unit variance scaling is applied to TF families. Both TFs and species are clustered using correlation distance and average linkage using ClustVis tool. (https://biit.cs.ut.ee/clustvis/). (a) Heat map of TFs reported earlier in published reports (CisBP, DBD and EupathDb) (b) Heat map representing distribution of TFs in the current version of ApicoTFdb.

Interestingly, the analysis also resulted in the identification of TF families which include TUB, NAC, BSD, HTH, Cupin/Jumonji, winged-helix and FHA families, not reported earlier for the above apicomplexans (Figure 2). GO-based TF-family assignments, integrated with manual curation, enabled inclusion of eight new TFs, which were missed during our previous conserved DBD domain analysis. As expected, a maximum number of TFs (*n =* 382) are from the AP2 family, followed by Zn-Finger, General-TF, Myb/SANT, FHA and HMG, with 237, 175, 122, 80 and 47 proteins, respectively, in the 14 apicomplexan species studied here (Table S4).

Our analysis also revealed that the FHA family is conserved across all the apicomplexans and over-represented in *Neospora caninum*. This family of TFs consists of a phosphopeptide-recognition domain and has been identified in eubacterial and eukaryotic genomes but unidentified in the archaeal genome. The FHA family is characterized by its multi-domain architecture in apicomplexans, which includes Zn-Finger C3HC4, prolyl isomerase (PPIC) and RNA recognition motif (RRM) along with the FHA domain. Apart from TFs, FHA family members include few phosphatases, kinases and RNA-binding proteins, which are involved in many different vital cellular processes (35).

**Figure 3:**
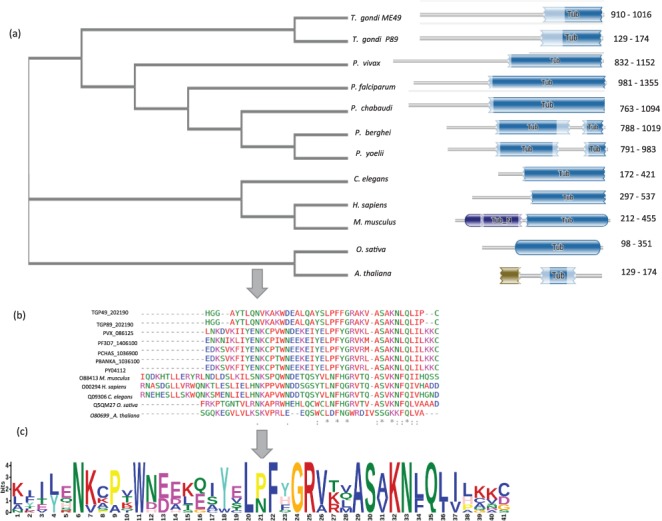
A schematic representation TUB family across 12 species shown here. (a) Phylogenetic analysis of TUB domain showing evolutionary conservation (using NJ method) sharing similar domain architecture (b) Conserved region across TUB domain among different species by multiple sequence alignment (using CLUSTAL-OMEGA) (c) Motif representation of TUB family proteins along with a consensus sequence logo across 12 species.

Another family of TF, not reported earlier for apicomplexans, is the TUB. TUB TFs play important roles in maintaining the functioning of neuronal cells during development and post-differentiation in humans, but till date they are not well reported in apicomplexans (36). Multiple sequence alignment revealed that TUB TF family members from model organisms harbor conserved Pfam domain PF01167 and have conserved a domain architecture in all the species, as shown in Figure 3.

Intriguingly, we observed that the BZIP and winged-helix TF families are not present in any of the six *Plasmodium* species.

### TcoFs prediction and classification

#### Classification of transcription-associated factors

**Figure 4:**
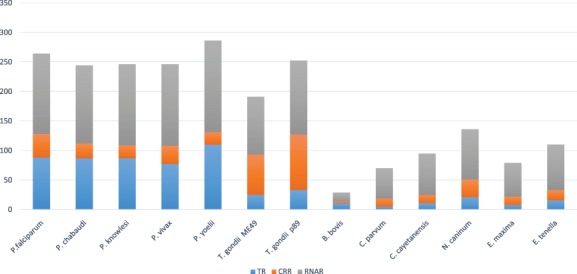
TcoFs distribution into TRs, CRRs and RNARs across apicomplexans.

The TcoF identification pipeline (see Section 2) predicted 2491 proteins. In order to remove the false-positive predictions (type I error) across different classes of TcoFs, manual curation on predicted TcoFs was performed. The curation resulted in removal of 1341 RNAR proteins with GO functions such as translation, tRNA processing, ribosome assembly and tRNA modification. Additionally, 125 proteins were found to show a GO-assigned function for both RNARs and TRs. For further classification, we manually analyzed each protein and classified accordingly in their classes into TRs, CRRs and RNARs. This resulted in identification and classification of 666 transcription regulators (TRs), 1412 RNARs and 415 chromatin regulators (CRRs), distributed among the 14 studied apicomplexan species. Among these, there are 637 TcoFs with annotation as hypothetical, conserved proteins with unknown function, unspecified product and conserved hypothetical proteins (Table S5). Figure 4 summarizes the species-wise distribution of TcoFs with most TcoFs observed in *Plasmodium yoelii*. To the best of the authors’ knowledge, this is a first-ever attempt to classify TcoFs into TRs, CRRs and RNARs in apicomplexans.

#### Web interface and annotations in ApicoTFdb

**Figure 5:**
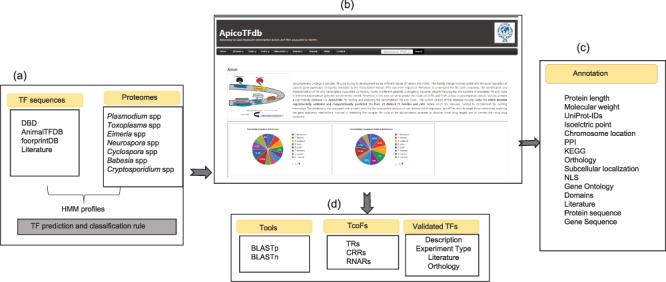
ApicoTFdb: Development and working pipeline (a) TF prediction and classification rule using known TF sequences. (b) Home page of ApicoTFdb is divided into different sections, namely browse, tools (includes BLASTP and BLASTn), TcoFs, other links, statistics, and tutorials (c) Annotation available in ApicoTFdb (d) TcoFs are mainly classified into 3 main classes i.e. TRs, CRRs and RNARs with their associated annotation and information related to experimentally verified TFs is also presented in a separate section.

**Figure 6:**
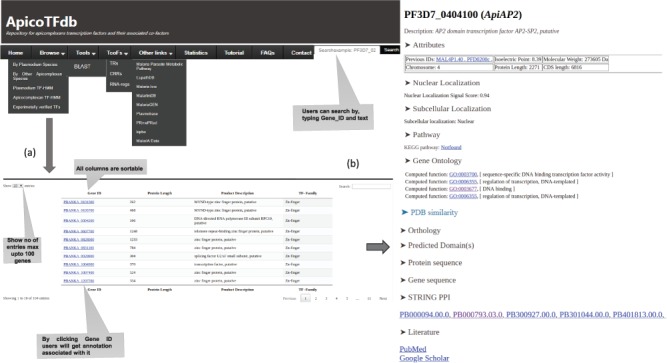
(a) ApicoTFdb home page defining each tab. (b) Per gene page and detailed annotation associated with each TF. Each TF is associated with annotations viz. Molecular weight, Protein length, UniProt Ids, Isoelectric Points, Protein-Protein interactions, KEGG pathway, Orthologous groups, Subcellular localization, Nuclear localization signals, Gene ontology, Domain information, Related literature and protein and gene sequences.

The ApicoTFdb project is presented as a novel web-based repository for apicomplexan TF and TcoF information retrieval as shown in Figure 5. The ApicoTFdb database project is organized according to the above described classification of TFs and TcoFs into different families based on intrinsic conserved domains and their respective gene ontologies. We included gene/protein-level information from several relevant web resources including EuPathDB, UniProt and Pfam for TF DBD Profile generation; OrthoMCL for orthology profiling; Gene Ontology using AMIGO, PubMed and Google Scholar for related literature information; KEGG (molecular pathway analysis) and STRING database for protein-protein interactions; and isoelectric point, molecular weight, CDS length and chromosome location, which provides necessary information, for apicomplexan TFs and TcoFs (as shown in Figure 6). We have also used CDD (37), PFAM (38), Superfamily (39) and SMART (40) for domain prediction; CELLO2GO for subcellular localization; PATS for apicoplast targeting sequences; and NCBI-BLAST, including BLAST*n* and BLAST*p*, for homology predictions.

#### Comparison with existing databases and validation

Only a limited number of databases provide information related to the predicted as well as experimentally validated apicomplexan TFs and TcoFs, e.g. DBD and CISBP. Though the database is useful, we observed that information available in CISBP (Database build 1.02) is outdated as it was implemented via the older version of PlasmoDB (version 10), is no longer in use and contains a large set of obsolete IDs. For instance, the database classifies PF14_0010 into the p53 domain, which has been changed to GBP_repeat in the current PlasmoDB annotation (PlasmoDB version 38). Another database viz. DBD with Pfam profile-based prediction also possesses limited capabilities and includes only a small set of TFs for the given apicomplexan species, e.g. only 18 TFs for *P. falciparum*. Table 1 summarizes the number of apicomplexan TFs reported in ApicoTFdb which compliments information given in other databases along with the unique set (Table 1**)**. In order to evaluate the confidence of putative transcription factors, we compared our prediction with the published reports (Table S6). Our findings revealed not only previously predicted and experimentally verified apicomplexans TFs but also other novel regulatory proteins not reported earlier.

## Discussion

Despite the profound role of transcription regulators in mediating the complex life cycle of apicomplexan parasite across multiple hosts, the number of known and putative regulators in these organisms is exceptionally low. Currently, repositories like EuPathDB are the prime source of putative or known TFs in the parasite genomes. However, most of the available genomes of these parasites are still incomplete and yet to be fully annotated with a large number of ‘hypothetical proteins’ and ‘proteins with unknown function’. Moreover, identification of proteins that can be classified under the TF/TcoF superfamily is still a daunting task. Thus, there is an urgent need of a dedicated data resource for retrieving known/putative transcription regulators within different parasite genomes. To identify key transcription regulators in apicomplexan species, we performed an exhaustive scrutinizing of the known DBDs across 14 parasite proteomes. Thereafter, we developed ApicoTFdb, the first exclusive web repository for hosting manually curated TFs and TcoFs identified in 14 apicomplexan proteomes.

Among the previously identified TFs, the AP2-family TFs are overrepresented in the parasite genomes studied here. Our extended analysis indicates that differences in the distribution of different classes of TFs and TcoFs exist across different genomes studied here. We are also successfully able to predict and classify TFs of genomes including *Babesia bovis*, *Eimeria maxima*, *Eimeria tenella*, *Cyclospora cayetanensis* and *Toxoplasma gondii* ME49 in which an extremely limited number of studies till date have been conducted to identify TFs. These species are the causative agent for the intestinal illness in humans, hemorrhagic cecal coccidiosis in young poultry, cattle fever and toxoplasmosis, a worldwide disease that infects one-third of the human population (41). Moreover, we observed that protein domain information alone is not sufficient to classify a protein under the TF or TcoF superfamily. Therefore, to restrict the type I error in our prediction, we classified a given protein under the TF or TcoF family only after the functional analysis and manual curation, which highlighted several unannotated proteins such as TF and TcoFs. For instance, several new TFs and TcoFs were identified which were earlier assigned as ‘hypothetical proteins’ or ‘proteins with unknown function’. Thus, the database provides a unique platform to illuminate the list of putative regulators, which otherwise remain obscure with existing portals.

### Future perspectives

We will be incorporating more apicomplexan species with their updated annotations. In the next update, we will provide another annotation associated with TFs which includes gene expression profile patterns, TF-binding motif identification and phylogenetic analysis. ApicoTFdb will be updated according to recent updates in the EuPathDB database.

ApicoTFdb will be useful to the researchers interested in less-studied gene regulatory mechanisms mediating the complex life cycle of the apicomplexan parasites. The database will aid the discovery of novel drug targets to much needed combat the growing drug resistance in the parasites.

## Supplementary Material

ApicTFdb-suppl_baz094

## Notes

**Database URL:** http://bioinfo.icgeb.res.in/PtDB/
